# Can epidemic detection systems at the hospital level complement regional surveillance networks: Case study with the influenza epidemic?

**DOI:** 10.1186/1471-2334-14-381

**Published:** 2014-07-10

**Authors:** Solweig Gerbier-Colomban, Véronique Potinet-Pagliaroli, Marie-Hélène Metzger

**Affiliations:** 1 Hospices Civils de Lyon, Hôpital de la Croix-Rousse, Unité d’hygiène et d’épidémiologie, F-69317 Lyon, France; 2 Université de Lyon, F-69000 Lyon; Université Lyon 1; CNRS, UMR5558, Laboratoire de Biométrie et Biologie Evolutive, F-69622 Villeurbanne, France; 3 Hospices Civils de Lyon, Hôpital de la Croix-Rousse, Service des urgences, F-69317 Lyon, France

**Keywords:** Emergency service, Hospital, Syndromic surveillance, Influenza, Human, Infection control, Disease outbreak, Population surveillance

## Abstract

**Background:**

Early knowledge of influenza outbreaks in the community allows local hospital healthcare workers to recognise the clinical signs of influenza in hospitalised patients and to apply effective precautions. The objective was to assess intra-hospital surveillance systems to detect earlier than regional surveillance systems influenza outbreaks in the community.

**Methods:**

Time series obtained from computerized medical data from patients who visited a French hospital emergency department (ED) between June 1st, 2007 and March 31st, 2011 for influenza, or were hospitalised for influenza or a respiratory syndrome after an ED visit, were compared to different regional series. Algorithms using CUSUM method were constructed to determine the epidemic detection threshold with the local data series. Sensitivity, specificity and mean timeliness were calculated to assess their performance to detect community outbreaks of influenza. A sensitivity analysis was conducted, excluding the year 2009, due to the particular epidemiological situation related to pandemic influenza this year.

**Results:**

The local series closely followed the seasonal trends reported by regional surveillance. The algorithms achieved a sensitivity of detection equal to 100% with series of patients hospitalised with respiratory syndrome (specificity ranging from 31.9 and 92.9% and mean timeliness from −58.3 to 20.3 days) and series of patients who consulted the ED for flu (specificity ranging from 84.3 to 93.2% and mean timeliness from −32.3 to 9.8 days). The algorithm with the best balance between specificity (87.7%) and mean timeliness (0.5 day) was obtained with series built by analysis of the ICD-10 codes assigned by physicians after ED consultation. Excluding the year 2009, the same series keeps the best performance with specificity equal to 95.7% and mean timeliness equal to −1.7 day.

**Conclusions:**

The implementation of an automatic surveillance system to detect patients with influenza or respiratory syndrome from computerized ED records could allow outbreak alerts at the intra-hospital level before the publication of regional data and could accelerate the implementation of preventive transmission-based precautions in hospital settings.

## Background

There are three categories of Transmission-Based Precautions: Contact Precautions, Droplet Precautions, and Airborne Precautions. Transmission-Based Precautions are used when the route (s) of transmission is (are) not completely interrupted using Standard Precautions alone [1]. In hospitals, knowledge of the admission of patients with potentially transmissible infectious diseases is important to institute appropriate infection control transmission-based precautions, without waiting for microbiological confirmation of the diagnosis. For example, nosocomial influenza outbreaks that began from a patient with community-acquired influenza have been described [2-4]. The rapid implementation of transmission-based precautions is a key factor in limiting the spread of microorganisms to other patients or to healthcare workers.

A surveillance system for detecting patients with potentially transmissible infectious diseases (UrgIndex) is being developed at the North Hospital Group of the Lyon University Hospitals (France). This system is based on the principle of the syndromic surveillance and is described in detail in other publications [5,6]. Briefly, it aims to detect automatically patients with potentially transmissible infectious disease, using data contained in the computerized medical records of the emergency department (ED). The detection of potentially infected patients has two objectives. The first objective is to identify individuals who consult in ED and are at epidemic risk for other patients and health care workers. Patients are classified by the surveillance system according to three syndromes: respiratory, cutaneous and gastrointestinal. This would alert infection control practitioners and healthcare workers and help them to set up the appropriate transmission-based precautions as soon as the patients’ care begins in ED, without expecting the diagnosis confirmation. The second objective of the system is to detect an increase in the number of patients who visit ED with potentially transmissible infectious diseases before other regional surveillance systems alert healthcare workers about outbreaks beginning in the community. Such alert would help infection control practitioners to make as soon as possible healthcare workers sensitive to the epidemic risk.

In the Rhône-Alpes region (France), there are various surveillance systems to detect the beginning of seasonal influenza outbreaks in the community.

For example, surveillance based on ED and mortality data (Surveillance sanitaire des urgences et des décès, SurSaUD®) is collected by the French national institute for public health surveillance (Institut de Veille Sanitaire, InVS), which depends on the Oscour® network (Organisation de la surveillance coordonnée des urgences). This system monitors the number of ED visits for influenza each week during the winter [7,8]. The French Sentinel network (Réseau Sentinelles, INSERM, UPMC) monitors influenza-like illness. These data are obtained from reports of medical consultations by network-participating physicians [9,10]. The GROG network (Groupes régionaux d’observation de la grippe, http://www.grog.org/) monitors the arrival and the circulation of influenza, and compares the circulating strain with those used to make the current seasonal vaccine against influenza [11]. The overall objective of those surveillance systems is to provide public health authorities with decision-making tools to allow them to remind the general public of the best practices to reduce the risk of inter-human transmission as soon as outbreaks emerge, and to organise necessary healthcare services. Another interesting data source for the detection of abnormal phenomena or epidemics in the community is the Google Flu Trends system. It monitors weekly patterns in Google searches on influenza to estimate flu activity [12,13]. The publication interval for surveillance results is short, and it is possible to receive the bulletin of the Sentinelles network and GROG weekly by email. However, a few days pass between the end of each weekly data collection period and the analysis and publication of the results. A few days’ delay in the transmission of information to health professionals can result in the transmission of an infection within a hospital if the transmission-based precautions were not applied.

The objective of this study was to evaluate whether intra-hospital surveillance systems including an automatic syndromic surveillance system can detect the onset of influenza epidemics earlier than regional influenza surveillance networks. That would permit earlier implementation of preventive measures that must be put in place throughout the hospital.

## Methods

### Hospital surveillance data

This study is not an experimental research but an observational and descriptive epidemiological study using anonymous data extracted retrospectively in our hospital database. In accordance with the French legislation, this type of analysis does not require approval by an ethics committee and the study is registered under No. 13–156 in the registry of our hospital for data processing exempted from declaration to the CNIL (Commission Nationale de l’Informatique et des Libertés, French Commission on Information Technology and Liberties).

Hospital surveillance data were extracted and processed from ED’s electronic medical record of patients older than 15 years of age who visited the adult ED of the North Hospital Group, Lyon University Hospitals, between June 1st, 2007 and March 31st, 2011 (N = 101,001). Three time series were constituted from hospital data: they were named UrgIndex-hospitalisations, ICD10-consultations, and ICD10-hospitalisations.

#### Syndromic surveillance based on the automatic extraction from medical records of the emergency department (UrgIndex-hospitalisations)

The first hospital time series, UrgIndex-hospitalisations, is based on the data extraction of the patient’s medical record. The ED computerized medical record is made up of two types of variables: structured variables (e.g., age, emergency diagnosis code), and variables related to medical writing in the medical record using natural language (e.g., chief complaint, observation notes). The data extracted from the ED computerized medical record were processed by UrgIndex. The corresponding algorithm includes the processing of two types of variables (structured and free text variables) and contains three steps:

1) matching keywords that describe different syndromes with the text of the computerized medical record, by means of an application that automatically processes natural language variables [5].

2) for each patient, calculating the probability of having a potentially transmissible infection and

3) determining whether the probability is above a detection threshold set by the user depending on the sensitivity and positive predictive value of the detection algorithm [6].

The time series was constituted of the daily number of patients hospitalised with respiratory syndrome after an emergency visit in the same hospital. The infectious diseases detected by UrgIndex and classified into “upper airways or respiratory syndrome” were influenza, viral pneumonias other than influenza, bacterial pneumonias, bronchitis, infections of the upper airways, and tuberculosis.

#### Influenza surveillance based on ICD-10 codes assigned by physicians after ED consultation (ICD10-consultations)

Discharge summaries, produced at the end of each visit in an ED, provide information necessary for regional and national health surveillances. In 2006, the content and format of this discharge summary were defined at the national level. Data to be transmitted to a regional server (Oscour server), must be extracted from computer systems deployed in the EDs. These data include the ICD-10 coding of the medical cause of the visit [14]. This coding is done by the emergency physician at the end of the consultation.

The second hospital time series (ICD10-consultations) were composed of the daily number of patients who visited the ED for a medical cause coded as influenza (J09, J10, J11, J10.0, J10.1, J10.8, J11.0, J11.1, and J11.8) in the discharge summary of the ED consultation.

#### Influenza surveillance based on ICD-10 codes assigned by physicians in patients hospitalised after ED consultation (ICD10-hospitalisation)

The third time series (ICD10-hospitalisation) were composed of the daily number of patients hospitalised after an ED visit and for whom a medical cause coded as influenza (J09, J10, J11, J10.0, J10.1, J10.8, J11.0, J11.1, and J11.8) in the discharge summary of the ED consultation.

### Regional surveillance data

Four time series were obtained using regional data from existing influenza surveillance systems.

#### Oscour® surveillance system

The first series included the daily number of patients who visited EDs for influenza in hospitals participating in the Oscour® network (Oscour-consultations). This network collects summary data extracted from computer systems deployed in the EDs and transmitted to the Oscour® regional server.

The second series included a subgroup of the first series: the daily number of patients hospitalised for influenza after an ED visit within the Oscour® network (Oscour-hospitalisation).

The Oscour® system started operating in June 2009 in the Rhône-Alpes region. The time series began on June 29th, 2009 (week 27) and ended on April 3rd, 2011 (week 13). The data analysed in this study came from 19 hospitals that participated in the network throughout the study period. The local hospital data presented in this study came from one of the nineteen participating hospitals. Discharge diagnostic codes were available for at least 70% of the patients at all but one hospital, for which it was only 10%.

#### Regional sentinel network

The second source for regional series was from the Sentinel network for the Rhône-Alpes region. The corresponding third series included the weekly number of patients who consulted their general practitioner for influenza-like illness (i.e., sudden fever > 39°C, with myalgia and respiratory signs). The data in this series were downloaded for the Rhône-Alpes region and the study period from the website of the Sentinel network, INSERM, UPMC (http://www.sentiweb.fr).

#### Google flu trends website

The fourth regional series included the weekly number of queries about influenza on the Google search engine that were made by users living in the Rhône-Alpes region. The data were downloaded for the study period from Google Flu Trends website (http://www.google.org/flutrends/fr/#FR).

### Descriptive analysis of local hospital and regional time series

Regional and local hospital data were aggregated by week and described graphically for seasonal fluctuations, amplitude of the fluctuations, and the beginning of peaks of activity. The total number of ED visits, hospitalisations or Google queries was calculated for every outbreak period and for every time series.

### Method to detect community outbreaks from local hospital data

The cumulative sum (CUSUM) method was applied to local hospital data for outbreak detection by calculating the numbers of daily patient visits and identifying those that were above an outbreak threshold (set by a computational method described below). The three algorithms described by Hutwagner for the Early Aberration Reporting System (EARS) surveillance system developed by the United States Centres for Disease Control and Prevention (CDC) were applied to the hospital time series [15]. The formula for the three algorithms was:

$$S_{t}=\text{max}\left[0;S_{t-1}+\frac{X_{t}-\left(\mu_{0}+k\sigma_{\mathrm{xt}}\right)}{\sigma_{\mathrm{xt}}}\right]$$

S_t_ is the CUSUM statistics computed at t-time, X_t_ the number of observations at t-time, μ_0_ the expected mean, σ_xt_ the variance, and k the detectable difference to the mean. The CUSUM algorithms used three different moving average calculation methods for μ_0_ (C1-mild, C2-medium, C3-ultra). The C1-mild calculation method used a moving average calculated as the mean of ED visits during the previous 7 days (t-7 to t-1), the C2-medium calculation method used a moving average calculated as the mean of visits during the previous 7 days with a free interval or lag of 2 days (t-9 to t-3), and the C3-ultra calculation method used a moving average calculated during the same period as C2, but the C3 statistic was the sum of the statistics from three sampling days, S_t_, S_t-1_, and S_t-2_). An algorithm was built for detecting a new outbreak into 3 steps:

1) St calculation using either C1, C2, C3 for estimation of μ_0_ and different k values.

2) Defining the threshold of outbreak detection: this threshold was determined as a value of μ_0_ (average number of visits) plus three standard deviations (SD). A signal was defined as a value of S_t_ exceeding this threshold.

3) Defining the duration of the signal for triggering an alert: different numbers of consecutive days with a signal to consider a potential outbreak community were assessed: one-day signal, 3-days signal and 5-days signal.

The variation of values of S_t_, k, and number of consecutives days, allowed to evaluate different algorithms. A total of 54 algorithms were assessed for the three time series.

### Evaluation of detection performance

For evaluating the ability to detect community influenza outbreaks with ED data of a single hospital, the hospital series were compared with regional data. The outbreak reference periods used were taken from publications of the regional Unit (Cire Rhône-Alpes) of the French Institute for Public health surveillance (InVS), which are used to inform regional healthcare facilities when the threshold of detection of regional flu epidemic is reached. The outbreak thresholds were based on the application of the Serfling method to the regional Sentinel network data [16]. As regional data are available as weekly aggregated data, the beginning of an outbreak was defined as the first day of the week. We considered as true alert only signals that began before the outbreak period and were ongoing during the outbreak period or signals that began during the outbreak period. If a signal began after the outbreak period (without signal during the period), we considered this signal as a false alarm and that the outbreak was not detected.

**Figure 1:**
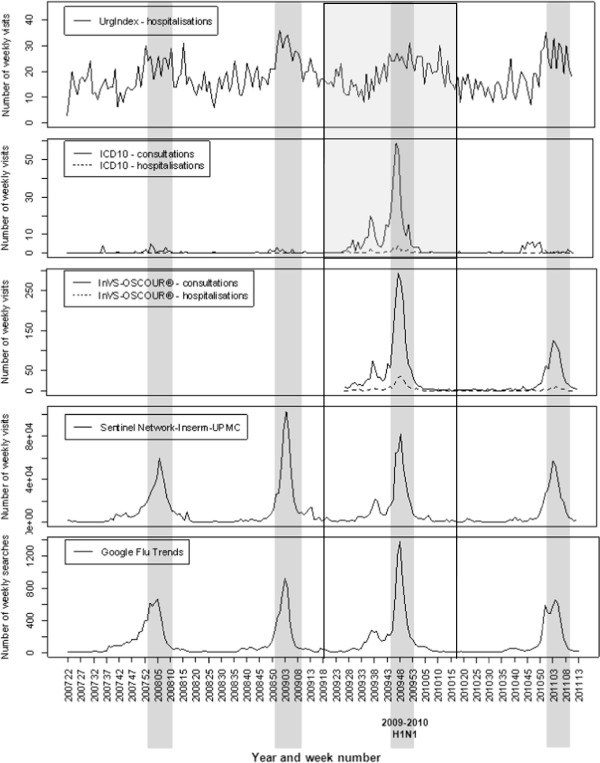
**Graphs of time series between 1st June 2007 and 31st March 2011.** Time series for respiratory syndromes detected in the local hospital by UrgIndex and the weekly influenza cases of the different surveillance systems studied. The shaded regions are the reference for the influenza outbreak periods in the Rhône-Alpes region defined in publications of the Cire Rhône-Alpes from regional Sentinel network data. Solid lines show the period of data excluded for the sensitivity analysis (from 2009-S19 to 2010-S18).

The algorithms’ performance was assessed using sensitivity, specificity and timeliness defined by Hutwagner et al. and Cowling and al [15,17]. Sensitivity was defined as the number of outbreaks in which ≥ 1 day was flagged by the CUSUM algorithm divided by the total number of outbreaks. The specificity was defined as the number of days of non-epidemic periods and that were not flagged by the CUSUM algorithm divided by the total number days of non-epidemic periods. The mean timeliness was defined as the mean number of complete days that occurred between the beginning of an outbreak and the first day the outbreak was flagged. If the surveillance system detects the outbreaks before the community alert, the timeliness would be negative, and if the surveillance system detects the outbreaks after the community alert, the timeliness would be positive.In order to take into consideration the particular epidemiological situation related to pandemic influenza in 2009, a sensitivity analysis was conducted. This sensitivity analysis consisted in excluding the year 2009, starting from the end of the monitoring period of the previous year (period ended in 2009-S18), i.e. from 2009-S19 to 2010-S18 (see Figure 1).

## Results

### Description of time series

The time series are shown in Figure 1. The shaded regions are the reference data for influenza outbreak periods in the Rhône-Alpes region [16].

The local hospital data showed globally the same seasonal trends as the regional data. The weekly number of ED visits for influenza in our hospital (ICD10-consultations) usually fluctuated between 0 and 6, even during outbreak periods, except during the global influenza A/H1N1 pandemic when 59 visits were recorded during one week (week 48 of 2009). The number of patients hospitalised for influenza after their ED visit (ICD10-hospitalisations) was also significantly larger during the H1N1 pandemic than during the seasonal outbreaks reported here, which generally reached no more than four patients a week. The number of respiratory syndromes detected by the UrgIndex-hospitalisations system was very variable from one week to the next. These weekly fluctuations were found to a lesser extent in the regional series.Oscour® data showed that the increases in the number of visits for influenza during outbreak periods were proportionately much larger than the increase in the number of hospitalisations for influenza after an ED visit (Figure 1). The same pattern was seen for the results of ICD10-consultations and ICD10-hospitalisations during outbreaks compared to non-outbreak periods.

The Sentinel network data show that, over the period of study, the 2008–2009 seasonal epidemics had the largest number of consultations for influenza-like illness, with more than 100,000 general practitioner’s visits during week 4 of 2009 and a total of 483,852 consultations during the period. It was also the longest duration (13 weeks from week 51 in 2008 to week 11 in 2009).

Google Flu Trends data show that the number of queries on the Google search engine closely followed the data fluctuations of the Sentinel network. The increase in the total number of queries concerning influenza during the H1N1 outbreak was more prominent than the increase in the number of general practitioner consultations for influenza-like illness in the Sentinel network.

### Performance of CUSUM algorithms to detect community influenza outbreaks using ED data of a single hospital

A number of 54 algorithms, all variants of C1-mild, C2-medium, and C3-ultra, were assessed for the three time series evaluated. Different algorithms have achieved a sensitivity of detection equal to 100% with ICD10-consultations (16 algorithms) and UrgIndex-hospitalisations series (12 algorithms). The algorithms have not achieved sensitivity equal to 100% with ICD10-hospitalisation series. This detection sensitivity did not exceed 50%. The Table 1 shows the results of algorithm assessment for those where the sensitivity was equal to 100%, i.e. those which were able to detect all outbreaks.

**Table 1:** Performance of different detection algorithms with sensitivity of 100% to detect influenza (UrgIndex-hospitalisation and ICD10-consultations)

**Time series**	**Detection algorithms**	**Outbreaks periods**	**Non-epidemic periods**	
		**Mean timeliness (days)**	**Number of days with false alarms N = 1094**	**Specificity (%)**
UrgIndex - hospitalisations	C2, k = 0.08, 1d	−7.5	327	70.1
	C2, k = 0.08, 3d	−3.3	226	79.3
	C2, k = 0.1, 1d	−15.3	247	77.4
	C2, k = 0.1, 3d	5.0	155	85.8
	C3, k = 0.08, 1d	−58.3	817	25.3
	C3, k = 0.08, 3d	20.3	739	32.4
	C3, k = 0.08, 5d	−13.3	589	46.2
	C3, k = 0.1, 1d	18.3	745	31.9
	C3, k = 0.1, 3d	−18.3	658	39.9
	C3, k = 0.1, 5d	−13.3	589	46.2
	C3, k = 0.5, 1d	0.5	230	79.0
	C3, k = 1, 1d	5.5	78	92.9
ICD10 – consultations	C1, k = 0.07, 1d	−18.3	151	86.2
	C1, k = 0.07, 3d	−11.5	127	88.6
	C1, k = 0.07, 5d	−7.8	115	89.5
	C1, k = 0.1, 1d	−18.3	148	86.5
	C1, k = 0.1, 3d	−7.8	122	88.4
	C2, k = 0.07, 1d	−19.8	147	86.6
	C2, k = 0.07, 3d	0.5	135	87.7
	C2, k = 0.07, 5d	2.5	125	88.6
	C2, k = 0.1, 1d	−13.5	143	86.9
	C2, k = 0.1, 3d	0.8	131	88.0
	C2, k = 0.1, 5d	2.8	121	88.9
	C3, k = 0.07, 1d	−32.3	172	84.3
	C3, k = 0.1, 1d	−32.3	168	84.6
	C3, k = 0.1, 3d	−15.8	151	86.2
	C3, k = 0.1, 5d	−11.3	154	87.0
	C3, k = 0.5, 1d	9.8	74	93.2

C_1_, C_2_, and C_3_ refer to the three different moving average calculations of CUSUM statistics (C_1_-mild, C_2_-medium, C_3_-ultra).

k is the detectable difference to the mean used to the calculation of CUSUM statistics.

Negative mean timeliness: first day signal before the outbreaks beginning, on average.

Positive mean timeliness: first day signal after the outbreaks beginning, on average.

Using UrgIndex-hospitalisations series, the mean timeliness of CUSUM algorithms that allowed the detection of all outbreaks periods ranged between −58.3 and 18.3 days. The corresponding specificity ranged from 25.3 to 92.9% (Table 1). Using ICD10-consultations series, the timeliness of CUSUM algorithms that allowed the detection of all outbreaks period ranged between −32.3 and 9.8 days, whereas the specificity ranged from 84.3 to 93.2%.

The sensitivity analysis which consisted in excluding the year 2009, showed better results for both types of series except for mean timeliness in the ICD-10 consultation-series. The mean timeliness of CUSUM algorithms using UrgIndex-hospitalisations series ranged between −10.7 and 14.3 days and the corresponding specificity ranged from 29.4 to 94.8% (Table 2). Using ICD10-consultations series, the timeliness of CUSUM algorithms ranged between −8.0 and 27 days, whereas the specificity ranged from 93.9 to 98.2%.

**Table 2:** Performance of different detection algorithms with sensitivity of 100% to detect influenza (UrgIndex-hospitalisation and ICD10-consultations), excluding data from 2009-S19 to 2010-S18

**Time series**	**Detection algorithms**	**Outbreaks periods**	**Non-epidemic periods**	
		**Mean timeliness (days)**	**Number of days with false alarms N = 793**	**Specificity (%)**
UrgIndex - hospitalisations	C2, k = 0.08, 1d	3.7	149	81.2
	C2, k = 0.08, 3d	8.7	75	90.5
	C2, k = 0.1, 1d	3.7	120	84.9
	C2, k = 0.1, 3d	14.3	56	92.9
	C3, k = 0.08, 1d	−10.7	560	29.4
	C3, k = 0.08, 3d	−8.7	497	37.3
	C3, k = 0.08, 5d	−6.7	446	43.8
	C3, k = 0.1, 1d	−8.3	511	35.6
	C3, k = 0.1, 3d	−6.3	440	44.5
	C3, k = 0.1, 5d	−0.3	384	51.6
	C3, k = 0.5, 1d	4.0	139	82.5
	C3, k = 0.5, 3d	6.0	78	90.2
	C3, k = 1, 1d	4.0	41	94.8
ICD10 – consultations	C1, k = 0.07, 1d	1.0	37	95.3
	C1, k = 0.07, 3d	5.0	23	97.1
	C1, k = 0.07, 5d	12.3	15	98.1
	C1, k = 0.1, 1d	1.0	36	95.5
	C1, k = 0.1, 3d	5.0	22	97.2
	C1, k = 0.1, 5d	12.3	14	98.2
	C2, k = 0.07, 1d	−1.7	34	95.7
	C2, k = 0.07, 3d	24.7	26	96.7
	C2, k = 0.07, 5d	26.7	18	97.7
	C2, k = 0.1, 1d	6.7	30	96.2
	C2, k = 0.1, 3d	25.0	22	97.2
	C2, k = 0.1, 5d	27.0	14	98.2
	C3, k = 0.07, 1d	−8.0	48	93.9
	C3, k = 0.07, 3d	3.0	40	95.0
	C3, k = 0.07, 5d	5.0	35	95.6
	C3, k = 0.1, 1d	−8.0	46	94.2
	C3, k = 0.1, 3d	3.0	36	95.5
	C3, k = 0.1, 5d	5.0	31	96.1
	C3, k = 0.5, 1d	8.3	26	96.7

C_1_, C_2_, and C_3_ refer to the three different moving average calculations of CUSUM statistics (C_1_-mild, C_2_-medium, C_3_-ultra).

k is the detectable difference to the mean used to the calculation of CUSUM statistics.

Negative mean timeliness: first day signal before the outbreaks beginning, on average.

Positive mean timeliness: first day signal after the outbreaks beginning, on average.

## Discussion

The study showed that over the evaluation period, which included four influenza seasons between 2007 and 2011, the surveillance data of the local hospital followed the seasonal trends indicated by the regional surveillance systems.

The two time series (UrgIndex-hospitalisations and ICD10-consultations) can detect all outbreaks exhaustively (sensitivity of 100%). However, according to the algorithm used, the timeliness and specificity vary. The best specificities found with UrgIndex-hospitalisations series (92.9% and 85.8%) were associated with too long mean timeliness (respectively 5.5 and 5.0 days) for having any interest in early outbreak detection at the regional level. This result is consistent with the source data since UrgIndex is a tool for syndromic detection of pneumonia or infections of the upper airways while the consultation data take into account only the diagnoses codes corresponding to the flu. The Urgindex hospitalisation system is not appropriate in its current development to detect regional outbreaks because its detection algorithms are oriented around syndromic surveillance to alert as soon as possible infection control practitioners on an epidemic risk regardless the causative organism.

For a sensitivity of 100%, the balance between specificity and mean timeliness of the series ICD10-consultation is better than that of UrgIndex. Two detection algorithms presented a specificity higher than 85% with a satisfactory mean timeliness: C2, k = 0.07 and 3 days (sensitivity: 100%, specificity: 87.7%, and mean timeliness = 0.5) and C2, k = 0.1 and 3 days (sensitivity: 100%, specificity: 88.0%, and mean timeliness = 0.8). However, this specificity was still too low to consider a practical use, due to frequent false alarms (around 1,1 times/week).

Another point was to take into consideration the particular epidemiological situation related to pandemic influenza in 2009, which could influence the results for detection of seasonal flu outbreaks. The same analysis was conducted with exclusion of the year 2009. Indeed, the performances were improved. The best balance between specificity and mean timeliness was obtained with ICD10-consultations series: C2, k = 0.07, 1d (sensitivity = 100%, specificity = 95.7%, and mean timeliness = −1.7 day). This performance would be satisfactory for a practical use, the number of false positives being around 1.5 per month.

In the interpretation of these results, it would be important to take into account that the 2009–2010 influenza season was particular because of the media coverage due to the H1N1 pandemics that began early in the spring 2009. The seasonal outbreaks were often detected by the algorithms more than 2 months before the community outbreak. The figure shows that in each surveillance system there was an increase in the number of cases before the outbreak period during spring 2009. Many patients consulted for influenza-like illnesses, but it was attributed to other circulating viruses. However, although those viruses were less pathogenic than *Myxovirus influenza*, they may lead to intra-hospital transmissions and need to be detected by our syndromic surveillance system (UrgIndex).

The graphs of the time series showed that for our hospital, during the pandemic seasons of H1N1 influenza, the number of ED visits for influenza was at least 20-fold greater than in the other seasons. The number of patients hospitalised for influenza during the pandemic was greater than in the other seasons, but not by the same proportion. Nevertheless, during the same period, the total number of patients who consulted general practitioners within the Sentinel network for influenza-like illness was lower than the number of consultations in the previous season. These observations suggest that the patients who had clinical signs of influenza during the H1N1 pandemic visited EDs rather than their general practitioner. This phenomenon seems to result more from the patient’s fear of the virus than from the actual gravity of the disease, as has previously been described [18-20]. This hypothesis is supported by the description of the time series of influenza queries on the Google engine, which were 1.5-times higher during the pandemic period than during the other seasons.

The study showed that the local data of ED visits agreed with the seasonal trends reported by regional surveillance systems and could allow earlier detection of community outbreaks of influenza at the local hospital level. The use of local data can permit better reactivity for the hospital to alert healthcare workers at the beginning of the outbreak. To set up such a system in a hospital, the targeted performances that can be recommended are a sensitivity of 100%, a specificity of 95% and timeliness close to 0 days. This choice is the result of a balance between detecting all outbreaks without having too many false positives alerts. A specificity of 95% was empirically chosen regarding the number of false alerts generated with this threshold at the intra-hospital level, corresponding to 2 false alerts per month in our regional epidemiological situation. In our study, these performances were achieved with the ICD10-consultation series when excluding the year 2009. However, it would be necessary to study the influence of diagnostic codes used for influenza regarding circulating influenza virus in the community, because Moore and *al*. demonstrated that knowledge of circulating influenza virus in the community strongly influences the diagnostic codes used when patients present to emergency departments with influenza-like-illness [21]. Nevertheless, it is important to precise that it is the doctors who perform the diagnostic coding in ICD-10 in our emergency department and not the clerical staff. Moreover, the aim of the system is to detect the flu outbreak earlier than the regional systems, then before medical awareness of the epidemiological situation. So, this type of bias should have more influence on the performances of the UrgIndex syndromic system than on ICD10-consultations series.

For this study, we used retrospective data of our hospital database because we aimed at evaluating the performance of these different series to complement the regional surveillance network. The results are encouraging for implementing this alert system in our hospital and could be developed when a generic Information Technology solution for extracting and analysing textual documents in the context of the daily activity of the hospital, will be available. The development of this solution (SYNODOS project) is in work progress and the solution could be available at the end of 2015 [22]. The reporting delay will be tested in this project but should be lower than 24 hours.

## Conclusions

Implementation of transmission-based precautions adapted to influenza (e.g., droplet precautions) could then be implemented when patients with clinical signs of influenza were admitted, without waiting for biologic confirmation. Indeed, the InVS warning system aims to detect unusual events at the national or regional level. The system does not primarily focus on communicating alerts at the intra-hospital level. The implementation of an intra-hospital warning system based on data from the ED computerized records would be complementary to regional and national surveillance systems.

## Competing interests

The authors declare that they have no competing interests.

## Authors’ contributions

SGC and MHM conceived the study. SGC built the algorithms, and performed the analyses. SGC, VPP, and MHM evaluated and determined which infectious diseases were important to detect. SGC, VP and MHM determined the algorithms to detect outbreaks. SGC drafted the manuscript, and MHM and VPP revised it. All authors have read, revised and approved the final version of the manuscript.

## Pre-publication history

The pre-publication history for this paper can be accessed here:

http://www.biomedcentral.com/1471-2334/14/381/prepub
